# Localized Temperature Variations in Laser-Irradiated Composites with Embedded Fiber Bragg Grating Sensors

**DOI:** 10.3390/s17020251

**Published:** 2017-01-27

**Authors:** R. Brian Jenkins, Peter Joyce, Deborah Mechtel

**Affiliations:** 1 Department of Electrical and Computer Engineering, US Naval Academy, 105 Maryland Ave, Annapolis, MD 21402, USA; mechtel@usna.edu; 2 Department of Mechanical Engineering, US Naval Academy, 590 Holloway Rd., Annapolis, MD 21402, USA; pjoyce@usna.edu

**Keywords:** fiber Bragg gratings, temperature sensors, polymer matrix composites, high energy radiation, strain sensors, structural health monitoring, smart structures

## Abstract

Fiber Bragg grating (FBG) temperature sensors are embedded in composites to detect localized temperature gradients resulting from high energy infrared laser radiation. The goal is to detect the presence of radiation on a composite structure as rapidly as possible and to identify its location, much the same way human skin senses heat. A secondary goal is to determine how a network of sensors can be optimized to detect thermal damage in laser-irradiated composite materials or structures. Initial tests are conducted on polymer matrix composites reinforced with either carbon or glass fiber with a single optical fiber embedded into each specimen. As many as three sensors in each optical fiber measure the temporal and spatial thermal response of the composite to high energy radiation incident on the surface. Additional tests use a 2 × 2 × 3 array of 12 sensors embedded in a carbon fiber/epoxy composite to simultaneously measure temperature variations at locations on the composite surface and through the thickness. Results indicate that FBGs can be used to rapidly detect temperature gradients in a composite and their location, even for a direct strike of laser radiation on a sensor, when high temperatures can cause a non-uniform thermal response and FBG decay.

## 1. Introduction

Optical fiber sensors provide a number of advantages for sensing either temperature or strain in polymer matrix composites. They are relatively nonintrusive, lightweight, and flexible. They respond rapidly, they can be used in harsh environments, and they are highly sensitive to both temperature and strain [1,2,3]. Fiber Bragg grating (FBG) sensors, in particular, can be used to accurately detect localized perturbations in strain and temperature with a high degree of spatial resolution due to their compact size [4,5]. This makes them especially attractive for structural health monitoring in polymer matrix composites to detect flames, radiation, or damage [6,7,8].

When used to measure high temperatures, FBGs may require appropriate annealing techniques or specialized fibers to prevent their decay or erasure [9,10,11,12]. For applications that simply require rapid detection of heat and its location on a composite surface, the high temperature decay of an FBG may be a secondary concern. For example, if an optical fiber sensor is used to detect flames [13] or to identify a directed energy assault [14] on an aircraft, the primary goal may be to simply recognize a spike in temperature as quickly as possible, similar to the way the human body responds to a burning sensation. The emphasis of this study is to determine the speed at which standard embedded FBG temperature sensors detect the temporal and spatial thermal response of a composite material or structure when high energy radiation is incident on the surface.

CO_2_ lasers emitting at 10.6 µm have been used in previous research to rapidly heat FBGs in bare fibers [15,16,17]. In this research, high energy infrared (IR) radiation at 1064 nm was used to heat a polymer matrix composite with embedded FBG temperature sensors. The thermal response of various composite test specimens was observed in this manner. Initially, two simple composite laminates were fabricated using E-glass fiber and carbon fiber with epoxy resin. A single optical fiber containing one to three sensors was embedded between two plies of each type of composite. The compact size and close spacing of the FBGs ensured high-spatial resolution temperature measurements. Additional tests were then performed on a more complex architecture, with 12 FBGs embedded in a 2 × 2 × 3 array in a two-ply carbon fiber/epoxy composite [18].

Though precise measurement of temperature is not essential in this application, reliable estimates of the temperature are still important to optimally design an FBG sensor network that can detect high-energy radiation, so an oven was used initially to calibrate the low temperature sensitivity of bare sensors and embedded sensors. Subsequent tests with a heat gun demonstrated the influence of conduction and convection on the thermal response in all of the composite structures [19]. In the tests described here, in which energy from a high power laser was absorbed by the composite, the primary mechanism of heat transfer was radiation from the laser strike, but conduction and convective cooling were also evident.

This study, using IR laser strikes at 1064 nm, demonstrated the feasibility of using networks of FBG temperature sensors to rapidly detect and locate radiation incident on the surface of a composite. The spatial response of the composite depended on the proximity of the sensor to the laser strike, whereas the temporal response was limited by the composite, not by the sensor. Development of an optimal sensor network and a detection algorithm that provides rapid and reliable detection of a thermal response will require additional research. Laser radiation directly incident on an embedded sensor caused a non-uniform temperature and strain profile [3,20], wavelength hopping due to peak wavelength interrogation at lower scan rates [21], and erasure (or partial erasure) of the grating at higher laser power levels. Because the sensors also measure any mechanical strain in the composite, interrogation of the full spectrum [5,22] may ultimately be required to fully identify and characterize the thermal and strain responses. Moreover, advanced signal processing may be needed to quickly isolate a laser-induced thermal response of an irradiated composite from mechanical strain.

## 2. Background

Physically, a fiber Bragg grating is a sinusoidal variation in the core index of refraction in an optical fiber along its longitudinal axis. The index of refraction is typically modulated using interferometric techniques to exploit the photosensitivity of optical fiber to ultraviolet radiation [23]. The most common technique used to manufacture an FBG uses phase masks to precisely control the physical characteristics of the grating.

For a grating period of Λ in a fiber with an effective index of refraction $n_{e},$ light propagating down the fiber at the Bragg wavelength $\lambda_{B}=2n_{e}\Lambda$ will be reflected by the grating. Light at all wavelengths other than $\lambda_{B}$ will continue to propagate beyond the grating with high transmission and low loss. Hence, an FBG can be used to selectively filter one (or more) wavelengths of the light propagating down the optical fiber.

Temperature sensing is possible because the Bragg wavelength shifts when the temperature changes. After taking the derivative of $\lambda_{B}$ with respect to temperature, the wavelength shift $\Delta \lambda_{B}$ that corresponds to temperature variation ΔT is:

(1) $$\Delta \lambda_{B}=\left(2\frac{dn_{e}}{dT}\Lambda +2n_{e}\frac{d\Lambda }{dT}\right)\Delta T.$$

Equation (1) is often normalized, such that:

(2) $$\frac{\Delta \lambda_{B}}{\lambda_{B}}=\left(\frac{1}{n_{e}}\frac{dn_{e}}{dT}+\alpha \right)\Delta T=6.67\times 10^{-6}\;{}^{\circ}C^{-1},$$

where the coefficient of thermal expansion of the optical fiber, α=1/Λ(dΛ/dT) is relatively small, contributing ~5% to the shift $\Delta \lambda_{B}$ [4]. Based on (2), the sensitivity to temperature $\Delta \lambda_{B}/\Delta T$ is near 10.3 pm/°C, at $\lambda_{B}=1550$ nm.

FBGs are also sensitive to strain, since any change in length due to tension (or compression) will lengthen (or shorten) the grating period. The normalized shift in Bragg wavelength due to applied mechanical strain ε is given by:

(3) $$\frac{\Delta \lambda_{B}}{\lambda_{B}}=\left\{1-\left(n_{e}^{2}/2\right)[P_{12}-\nu (P_{11}+P_{12})]\right\}\varepsilon =(1-p_{e})\varepsilon ,$$

where $P_{ij}$ are Pockel’s coefficients and ν is Poisson’s ratio [4]. The quantity $1-p_{e}\approx 0.78\times 10^{-6}{\mu \varepsilon }^{-1},$ so the sensitivity to strain at $\lambda_{B}=1550$ nm is 1.21 pm/µε. Strain may influence a temperature measurement if an FBG is intended for use purely as a temperature sensor.

If an FBG is embedded in a structure, (1) and (2) must be modified to account for strain (thermal or mechanical) in the composite. To consider both temperature and strain, (2) and (3) are combined, and the total normalized shift in $\lambda_{B}$ is given by:

(4) $$\frac{\Delta \lambda_{B}}{\lambda_{B}}=\left(\frac{1}{n_{e}}\frac{dn_{e}}{dT}+\alpha \right)\Delta T+(1-p_{e})(\varepsilon +\varepsilon_{t}),$$

where $\Delta \lambda_{B}$, due to thermal strain in the composite $\varepsilon_{t}=\alpha_{c}\Delta T$ depends on the coefficient of thermal expansion of the composite $\alpha_{c}$.

Before performing experiments using FBGs to detect localized temperature variations in a composite caused by laser irradiation, Equations (2) and (4) were experimentally characterized for the sensors and composites used in this project [19]. Type I FBG [9] temperature sensors from ITF Technologies were used in all tests, with nominal reflectivity values near 70% at $\lambda_{B}$. The sensor fibers were coated with polyimide, except for a 3–5 cm region around the FBGs where no recoating was applied. The vendor-specified maximum temperature was 300 °C. An oven was used to calibrate the sensitivity of both bare and embedded sensors to temperatures as high as 250 °C. In the preliminary characterization [19], the wavelength shift versus temperature in bare fiber was in good agreement with manufacturer specifications [24]. The resulting sensitivity $\Delta \lambda_{B}/\Delta T$ was 11.9% ± 5% pm/°C in the measured temperature range, both for sensors in bare fiber and for sensors embedded in the carbon fiber/epoxy composite. Hence, the carbon fiber composite exhibited a near-zero value for $\alpha_{c}$ such that $\varepsilon_{t}$ in Equation (4) was negligible; sensors embedded in the carbon fiber composite experienced minimal tensile or compressive strain as the temperature changed. In contrast, the sensitivity of sensors embedded in E-glass fiber/epoxy composite was 19.1 pm/°C. Based on the definition of $\varepsilon_{t}$ in Equation (4), $\alpha_{c}\approx$ 6 µε/°C in the E-glass fiber composite. The values for $\alpha_{c}$ in each material are consistent with another work ([25], pp. 375, 377). Thus, for a given temperature shift, the wavelength shift $\Delta \lambda_{B}$ in an E-glass/epoxy composite was expected to be >50% larger than that in a carbon fiber/epoxy composite.

**Figure 1:**
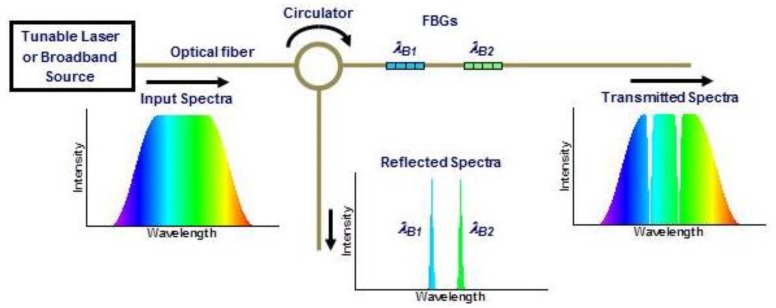
Typical sensor interrogation scheme for two inline fiber Bragg gratings (FBGs) with Bragg wavelengths $\lambda_{B1}$ and $\lambda_{B2}$.

To measure shifts in $\lambda_{B}$, an interrogation scheme similar to that shown in Figure 1 was employed. A tunable laser source was used in combination with a circulator to generate a reflected spectrum with energy at the Bragg wavelengths of all FBGs in the fiber. Shifts in the Bragg wavelengths were monitored at the reflected output port. Though only two sensors are illustrated in Figure 1, more were sometimes interrogated, depending on the size of the FBG array. The SmartScan interrogator (from Smart Fibres) used here could scan a 40-nm spectrum in four fiber optic channels at speeds up to 2.5 kHz with a spectral resolution around 0.1 nm, determining the peak wavelength of all the sensors every 400 µs. While the SmartScan interrogator scanned the spectrum at multi-kHz rates, the time required to detect a temperature gradient is intrinsically limited by the response time of the FBG sensor itself (i.e., when not embedded). Precise measurements of the sensor response time were not taken, though as a rough estimate, the heating response time appeared to be ~50 ms, with a somewhat slower cooling response [19], similar to results reported elsewhere [15,16,17].

**Figure 2:**
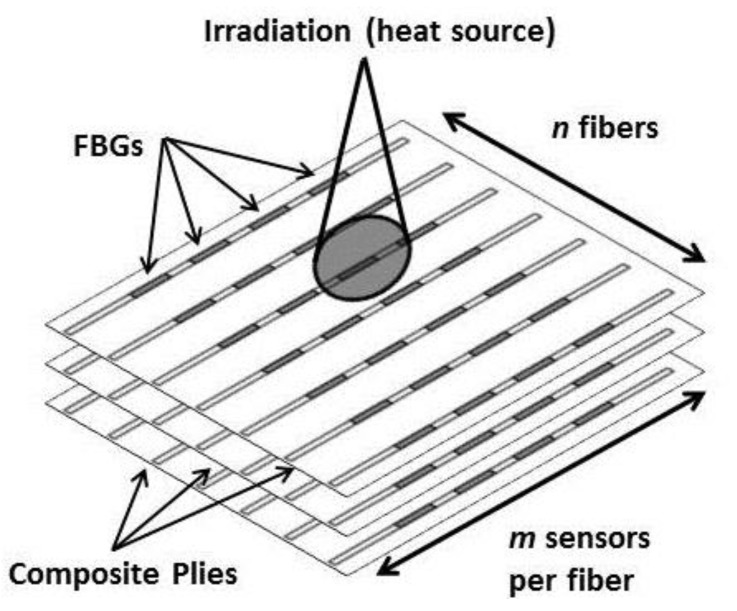
Multiple plies of composite with embedded n×m arrays of sensors on each layer.

In this research, the goal was to use FBG temperature sensors to rapidly detect and locate a temperature gradient in a laser-irradiated polymer matrix composite. However, as Equation (4) shows, $\lambda_{B}$ can shift either because of external strain ε or a change in temperature ΔT. An embedded sensor architecture that could be used to isolate temperature from strain is shown in Figure 2, where an n×m array of FBGs is embedded on each ply of the composite and laser irradiation is incident on a localized region on the surface. If external strain ε is uniform or predictable across the composite structure, it may be possible to isolate predictable shifts in $\lambda_{B}$ due to strain from localized shifts due to a temperature variation ΔT detected by only a few sensors.

For the tests performed here, the optical fibers were embedded on the surface or between the plies of the composite. The initial experiments focused only on the material response to the laser irradiation and did not include external strain, so ε=0 in Equation (4). While precise measurement of temperature is not the ultimate goal, estimates of the temperature will be helpful to diagnose and characterize the system response, especially at high temperatures. Ultimately, rapid detection of a localized temperature shift may require an interrogator with sufficient memory to capture the full spectrum and high speed signal processing [5,21,22] in order to isolate the thermal and mechanical responses due to temperature shifts and strain.

## 3. Localized Embedded Sensor Response

**Figure 3:**
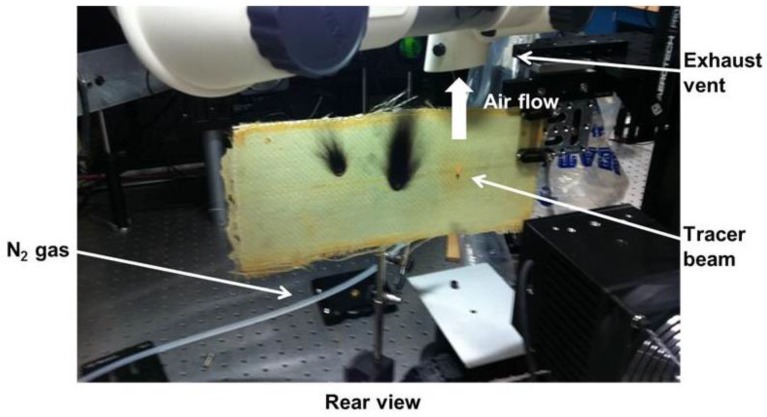
Test setup with the high power laser to measure the localized response of the embedded sensors to high temperature.

To observe the response of embedded temperature sensors to laser irradiation, a class IV laser was used to generate rapid heating at specific locations on the surface of each composite specimen. The laser emitted CW (continuous wave) light at 1064 nm with a beam diameter of 5 mm and variable output power up to ~100 W with a maximum irradiance of ~500 W/cm^2^. Figure 3 shows the rear view of the test setup with an E-glass fiber composite as the test article. An exhaust vent pulled air over the specimen to remove smoke from the chamber. N_2_ gas was applied as necessary to extinguish flames that erupted. For initial tests [19], the laser power was set to 25% of its maximum power, corresponding to ~25 W of total integrated power across the Gaussian beam profile. To measure the composite response over time, the interrogator scanned the spectrum at 500 Hz, acquiring the peak wavelength of each sensor every 2 ms. The initial tests described below considered a 70 s time window, beginning at *t* = 0 s. The laser was turned on at *t* = 10 s and off at *t* = 30 s in each test.

**Figure 4:**
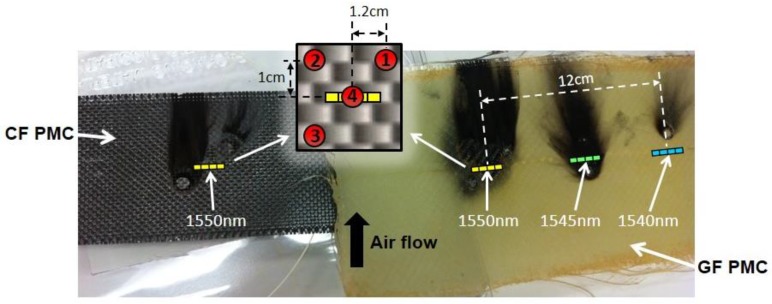
Polymer matrix composite (PMC) specimens tested for their high temperature response. Sensor positions in each PMC are indicated by their nominal Bragg wavelength. Air flow to remove smoke was upwards across each composite. CF: carbon fiber GF: E-glass fiber.

Figure 4 shows a picture of the two composite specimens after the initial tests were completed. Both specimens were manufactured with room temperature curing epoxy resin using either plain weave E-glass fiber (GF) fabric or carbon fiber (CF) fabric. The legend in Figure 4 identifies the locations of four laser strikes at positions 1–4 around each 1550 nm (nominal Bragg wavelength) sensor that was embedded in each composite. Direct strikes on the 1550-nm sensor in each specimen occurred at position 4. On the GF composite, two additional sensors were located 6 cm and 12 cm to the right of position 4, having nominal Bragg wavelengths of 1545 nm and 1540 nm, respectively.

Each FBG sensor is 1 cm in length. On each specimen, positions 1 and 2 are located 1 cm above, and 1.2 cm to the right and to the left of the 1550-nm sensor, respectively. Position 3 is located 1 cm below and 1.2 cm to the left of the sensor. The damage that occurred at each location is visible unless obscured by char from later tests, e.g., position 2 is obscured on the CF composite (char from previous experiments is also seen near the 1545-nm and 1540-nm sensors in the GF composite.)

**Figure 5:**
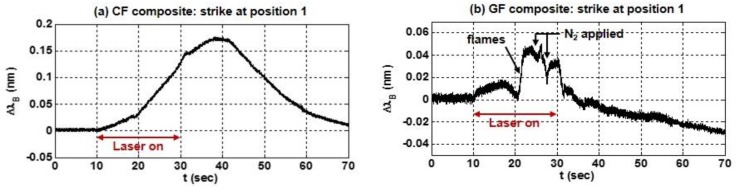
The temporal response of $\Delta \lambda_{B}$ during a strike at position 1, measured by the 1550-nm sensor in (**a**) the CF composite or (**b**) the GF composite.

Several general observations can be made based on the thermal response of the composites during the laser strikes at the locations shown in Figure 4. First, consider the laser strike at position 1. The responses measured by the 1550-nm sensors in each composite (1 cm below and 1.2 cm to the left of the strike) are shown in Figure 5. The nitrogen gas was used intermittently to extinguish flames that erupted from the composite surface as the epoxy resin in the composite laminate burned off. As seen in both plots, each composite responded immediately when the laser turned on at *t* = 10 s. Since the laser strike was above the sensor and the exhaust air flow (and N_2_) was upwards across the surface of each specimen, the shift in the Bragg wavelength and the temporal response depended primarily on the thermal absorptivity and thermal conductivity of the composite. For example, the higher thermal conductivity of the CF composite is evident in Figure 5a since the Bragg wavelength continued to shift upwards after the laser strike ended at *t* = 30 s; the measured temperature (detected by the sensor at position 4, below the strike at position 1) continued to increase as laser energy absorbed by the composite at position 1 continued to conduct past the FBG after the laser strike ended.

Furthermore, the magnitude of $\Delta \lambda_{B}$ in the CF composite in Figure 5a was larger than the shift in the GF composite in Figure 5b since the thermal absorption of the carbon fiber was greater than that of the E-glass fiber. Since the carbon is opaque and the E-glass is translucent, more energy was absorbed by the CF composite during the extent of the laser strike. This is consistent with measurements from a thermopile that was placed at the rear face of each specimen. No energy was measured by the thermopile on the rear side of the CF composite during the test, and though the laser strike did not burn a hole through either specimen during the tests, energy was measured exiting the rear face of the GF composite soon after the laser was turned on. The wavelength shift of 175 pm in Figure 5a indicates that the CF composite heated by approximately 15 °C, based on a sensitivity of 11.9 pm/°C. The peak temperature occurred near *t* = 38 s, ~8 s after the strike ended. In contrast, the GF composite temperature only increased by a couple degrees, as indicated in Figure 5b by a smaller shift of ~40 pm, using a sensitivity of 19.1 pm/°C. As observed in Figure 5b, the largest temperature increase in the E-glass occurred near *t* = 21 s, when flames erupted from the surface.

**Figure 6:**
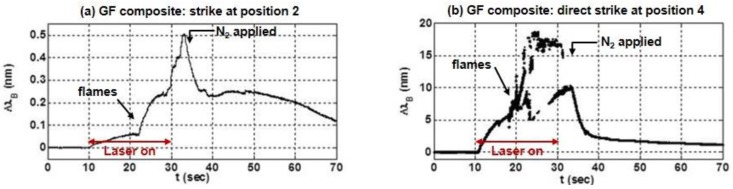
The variation in the 1550-nm sensor in the GF composite during laser strikes at (**a**) position 2 and (**b**) position 4.

The thermal response also depended on when (or whether) N_2_ gas was applied to the surface of the composite. This is observed in Figure 5b during the strike at position 1 when puffs of N_2_ were briefly applied to the surface of the GF composite near *t* = 25 s and *t* = 27 s. This was also evident during strikes at positions 2 and 4 on the GF composite, as shown in Figure 6 (a and b, respectively), which show the response of the 1550-nm sensor. During each of these strikes, nitrogen was not applied until *t* = 33 s, ~3 s after the laser turned off.

**Figure 7:**
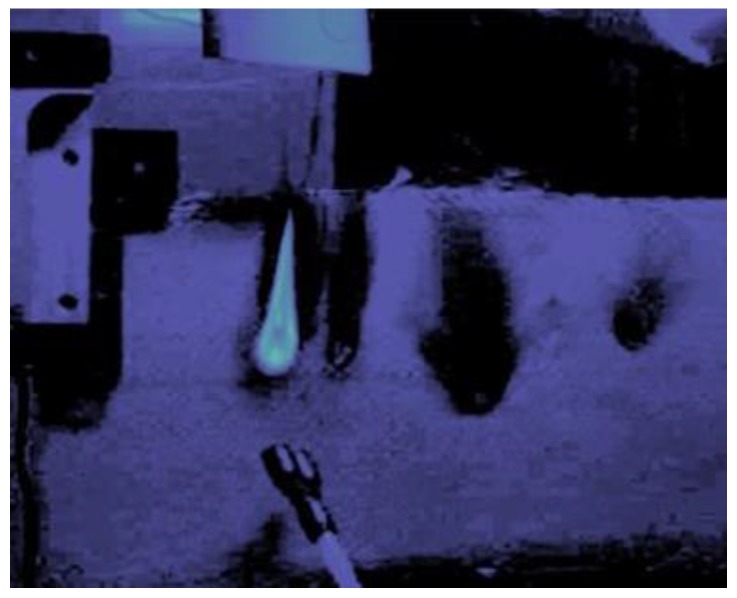
Flames erupting from the GF composite at *t* ≈ 30 s during a direct laser strike on the sensor at position 4. The N_2_ nozzle can be seen just below the flame.

The responses in Figure 6a,b also highlight the distinction between temperature changes due to heating from absorption of radiation, conduction or combustion, and convective cooling (using the N_2_). The large shifts in $\Delta \lambda_{B}$ that occur near *t* = 21 s in each plot coincide with the presence of flames on the surface during each strike, and the magnitude of $\Delta \lambda_{B}$ in these plots indicates that the 1550-nm sensor in the GF composite eventually achieved temperatures at least 10 times hotter than observed in Figure 5b, when the nitrogen gas was applied earlier, suppressing or extinguishing any combustion. As expected, the temperature increase was highest during a direct laser strike, as depicted by the erratic, fluctuating wavelength in Figure 6b. Figure 7 shows the flames erupting from the GF composite at position 4 near time *t* = 30 s. When the nitrogen was applied at *t* = 33 s, the combustion was extinguished and the temperature rapidly dropped.

The sensor in the GF based composite was severely damaged by the direct strike at position 4, as indicated by a significant drop in the optical power reflected back to the interrogator by the 1550-nm sensor, ~65% before the strike and ~8% after the strike. Based on the square law analysis and fiber characteristics in [12], such a partial erasure of the grating would require a 20-s exposure at a temperature of ~700 °C (well above the manufacturer specified operating limit of 300 °C for these sensors). For the FBGs used here, the sensitivity is nonlinear; a second order extrapolation to higher temperatures using data provided by the vendor [24] predicts wavelength shifts of at least 11 nm in bare fiber at 700 °C. Using the previous estimate of $\alpha_{c}\approx 6$ µε/°C in the E-glass fiber/epoxy composite, a total shift greater than 16 nm might be expected for temperatures near 700 °C when measured by a sensor embedded in the GF composite.

While a shift of this magnitude is somewhat consistent with the results in Figure 6b, the temperature response during a direct laser strike was difficult to determine precisely because of the reduced reflectivity in the spectral peak at $\lambda_{B}$. The peak wavelength measured by the interrogator was inconsistent (note the discontinuities in the plot); sometimes the peak was near the upper instrument limit of the interrogator at 1568 nm (a shift of nearly 18 nm) and sometimes the peak shifted between 5 nm and 10 nm. The SmartScan interrogator also occasionally dropped out completely without acquiring a peak wavelength. This phenomenon will be discussed in much greater detail later.

**Figure 8:**
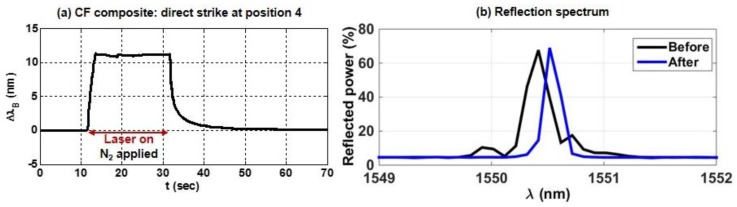
The (**a**) shift $\Delta \lambda_{B}$ and (**b**) the reflection spectrum (before and after) for a direct strike at position 4 in the CF composite.

For the CF composite specimen (see Figure 4), the results of a direct strike on the sensor are illustrated in Figure 8. The plot in Figure 8a shows the sensor response over time, and Figure 8b shows the reflection spectrum of the sensor before and after the laser strike. In contrast to the tests on the GF composite depicted in Figure 6 and Figure 7, the nitrogen was applied to the CF composite during the entire test to suppress combustion, and the thermal response was much more controlled. The sensor in the CF composite was very hot, given that the wavelength shift was greater than 10 nm. Furthermore, the response was very fast; the Bragg wavelength shifted by more than 10 nm in 1.5 s, with an estimated temperature shift of ~200 °C within the first 200 ms. Surprisingly, the FBG was not damaged during this test. In fact, as the epoxy burned off during the test the spectral response of the sensor was apparently restored (i.e., similar to its response in bare fiber before it was embedded in the CF composite). This is illustrated by the plot in Figure 8b. Embedding the sensors in the composite distorted the spectrum (illustrated by sidelobes in the black curve, not present in the original reflection spectrum for the sensor), but as the epoxy burned off, the reflection spectrum was restored to its initial apodized state (no sidelobes in the blue curve) [26], with slightly higher reflected power and a small increase in the Bragg wavelength, likely due to the release of a small residual compressive strain present after the sensor was embedded.

## 4. Advanced Sensor Network Response

**Figure 9:**
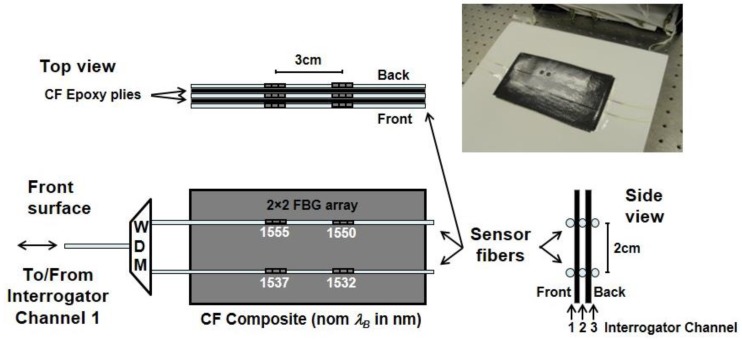
Test setup used to measure the response of a 2 × 2 × 3 array of sensors embedded on the surface and between two plies of CF composite. The nominal Bragg wavelengths of the FBGs are as shown. The actual composite specimen is shown in the upper right.

The results in Figure 6b and Figure 8 indicate that a large temperature shift could be quickly detected during a direct laser strike on a single sensor embedded in a composite. To more precisely determine how sensor location relates to the speed of detection, a more complex sensor network similar to Figure 2 was constructed to obtain multiple sensor measurements in the plane and through the thickness of a CF composite. Figure 9 shows the structure. The configuration uses a 2 × 2 × 3 array of 12 sensors in six optical fibers, with four sensors embedded on each surface, as well as between two plies of CF composite. As illustrated, two pairs of fibers are embedded on the front and back surfaces of the two-ply CF composite structure (see front surface view). A third pair of fibers is embedded between the two plies (see side view). The two fibers embedded at each layer are 2 cm apart, and the two sensors in each fiber are 1 cm in length, separated by 3 cm on centers. Three wavelength division multiplexers (WDM) route signals to/from the two fibers on each surface to communicate with three channels of the interrogator. The CF composite was fabricated by vacuum bag molding using West System^®^ 105/206 Epoxy Resin and 3 K Plain Weave Carbon Fiber fabric (5.7 oz./sq.yd, 0.012” thick, 12.5 × 12.5).

**Figure 10:**
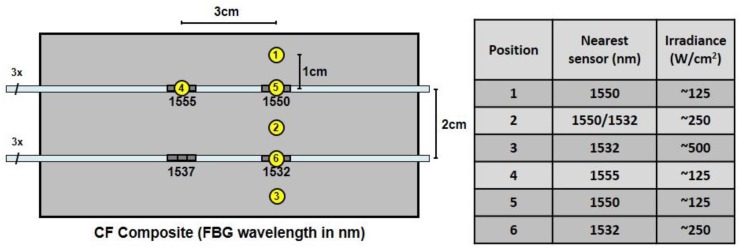
Locations and test conditions for six laser strikes on CF composite structure from Figure 9.

To determine how well an array of sensors can isolate a localized temperature gradient, the high power laser energy was applied to the front surface of the specimen in Figure 9 using the same test setup as in Figure 3. As before, the laser was on for 20 s during each strike. The scan rate of the peak interrogator was 2.5 kHz during these tests, so the peak wavelength of each sensor was determined every 400 µs. Numerous tests were run, and the laser was used to assault the composite at many different locations. Figure 10 shows a representative sampling of positions and test conditions for six laser strikes. The strikes at positions 1–3 were all 1 cm away from the nearest sensor at locations above, in between, and below the 1550-nm and 1532-nm sensors. The tests at positions 4–6 were direct strikes on the 1555-nm, 1550-nm and 1532-nm sensors, respectively. As before, the beam diameter of the laser was 5 mm and the maximum irradiance at 100% power was ~500 W/cm^2^. The power level was set to 25% of maximum at positions 1, 4 and 5, 50% of maximum at positions 2 and 6, and 100% at position 3. N_2_ gas was applied at each position during the full extent of each strike to prevent flames from erupting.

### 4.1. Response of 2 × 2 × 3 Embedded Sensor Array to Indirect Laser Irradiation

**Figure 11:**
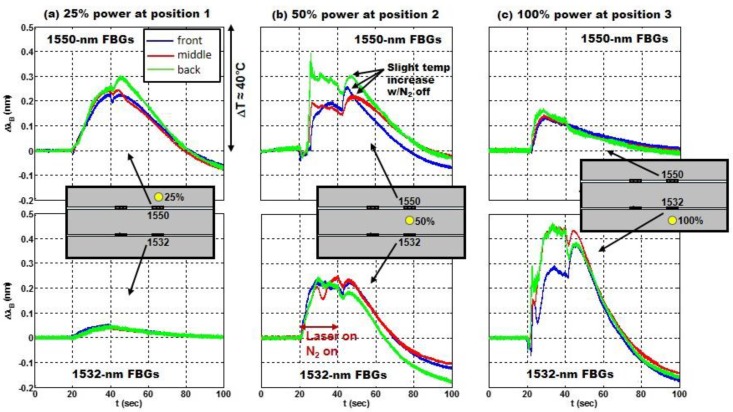
(**a**–**c**) The shift $\Delta \lambda_{B}$ vs. time for the FBGs with nominal Bragg wavelengths of 1550 nm and 1532 nm during laser strikes at positions 1–3, respectively, in Figure 10. The laser (and N_2_ gas) turned on at 20 s and off at 40 s. The specimen was oriented with air flow from right to left in strikes 1–3. The location of the strike, the relative laser power level and the corresponding FBG are shown in the inset for each pair of graphs.

The peak interrogator responses of the three 1550-nm sensors and the three 1532-nm sensors during the laser strikes at positions 1–3 are shown in Figure 11a–c, respectively. Based on a sensitivity of 11.9 nm/°C for sensors embedded in the CF composite, a shift of $\Delta \lambda_{B}=0.5$ nm on the vertical axis in Figure 11 corresponds to a temperature shift of just over 40 °C. In each test, the laser turned on at 20 s and off at 40 s. The responses of the sensors embedded on the front surface of the composite are shown in blue. The responses of the sensors embedded in the middle between the two plies of the composite are shown in red, and the sensors embedded on the back respond as shown in green.

Figure 11 illustrates both the effects of radial distance and laser power on the thermal response of the FBG sensors. The closer the sensor is to the laser strike and the greater the laser power the more the Bragg wavelength shifts. In Figure 11a the 1550-nm sensors exhibit a greater shift than the 1532-nm sensors because they are closer to the laser strike. In Figure 11c the laser power is increased from 25% to 100% and the Bragg wavelength shift increases irrespective of the sensor location relative to the laser strike, in this case the 1532 sensors are closer to the laser strike and exhibit a greater response. The sensors at 1555 nm and 1537 nm were embedded ~3–4 cm away from the laser strikes at positions 1–3 (see Figure 10). Their Bragg wavelength shift, not shown here, was relatively small, due to relatively poor thermal conductivity of the CF composite, confirming the localized thermal response to a laser strike and the ability to identify its location. Figure 11b shows the Bragg wavelength shift for a laser strike at 50% power and equidistant to the 1532-nm and 1550-nm sensors. The responses of the FBGs on the front surface and in the middle are very similar.

Since rapid detection of a laser strike is paramount, the speed of response of the FBG sensors may be of greater concern than either the fidelity of the temperature measurements or even the location information. The results in Figure 11 clearly show speed of response is dependent both on the power of the laser strike as well as the proximity of the sensor to the laser strike. Only 2 s are required to detect a 10 °C temperature shift in the 1532-nm sensor at 100% power (in Figure 11c), whereas for the same proximity and 25% power it takes nearly 8 s after the laser strikes the target to effect a 10 °C increase in the 1550-nm sensor (Figure 11a). Figure 11 (a and c) also clearly shows a steeper shift in the Bragg wavelength the closer the sensor is to the laser strike.

The previous results in Figure 6 in the GF composite show the presence of flames (combustion) and air flow (convection) could also impact the speed (and magnitude) of the response. In more than one instance Figure 11 shows the Bragg wavelength shift or thermal response of the sensor on the back surface (green) is greater or more erratic than that of both the front and the middle (blue and red). Flames were sometimes observed erupting from the back surface of the composite during a strike. The flames appeared like a “blow-torch” projected away from the back of the composite, in contrast to the appearance of the flame that was directed upward by natural convection in Figure 7. Whereas combustion was suppressed on the front surface using the N_2_ gas nothing was done to extinguish the flames on the back surface of the composite.

Heat transfer by conduction can be observed in Figure 11a–c. When the laser was turned off so was the N_2_ gas. Because the sensors in each case are a small distance from the laser strike, the heat of the laser strike is observed to diffuse to the sensor location; this is most evident in the slight temperature increase exhibited by the 1532-nm sensors at 40 s in Figure 11c. The temperature increase lasted for only ~5 s after the laser (and N_2_) was turned off. For these sensors the final wavelength was also less than the initial wavelength, likely due to relaxation of residual strain in the composite. Thru-thickness conduction is also evident in the 1532-nm sensors as the composite returns to thermal equilibrium; observe the temperature rise on the front surface once the N_2_ gas is turned off (Figure 11c).

**Figure 12:**
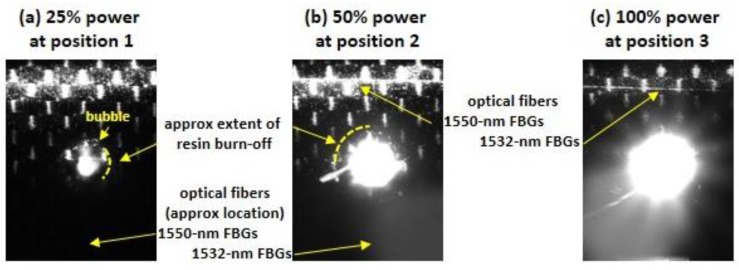
Video frames of the front of the CF composite, captured halfway through the strikes at positions 1–3, respectively. N_2_ gas flowed from right to left in each video frame.

Likewise, in video taken during the tests with N_2_ gas, no flames were observed on the front surface. Figure 12 shows three single frames of the video of the front surface captured around *t* = 30 s during the three laser strikes at positions 1–3, respectively. Few flames were observed on the front surface of the composite because they were extinguished by the nitrogen. However, the increasing irradiance associated with each strike is evident, as indicated by progressively larger “hot-spots” at positions 2 and 3. The tows of the carbon fiber fabric in the composite, ~2 mm in width, can be discerned in each frame. The optical fiber 1 cm above the strike at positions 2 and 3 can also be seen. Bubbles of epoxy were also occasionally observed sizzling and burning off at the edge of a circular region that slowly expanded in diameter during the strike.

### 4.2. Response of 2 × 2 × 3 Embedded Sensor Array to Direct Laser Strikes

**Figure 13:**
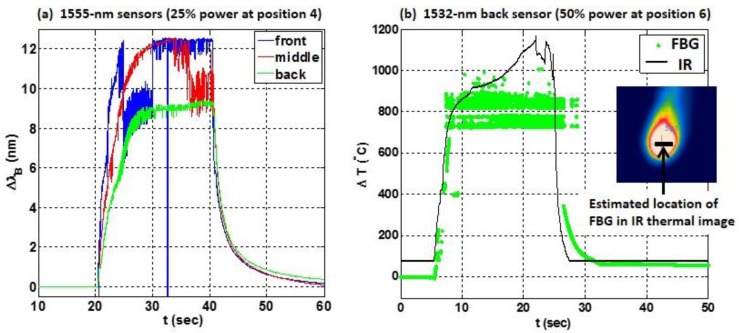
(**a**) Wavelength shift during a direct strike at position 4 in the front (blue), middle (red) and back (green) 1555-nm sensors (as shown in Figure 9); (**b**) Temperature on the back surface of the composite during a direct strike at position 6, estimated using the wavelength shift of the back 1532-nm sensor (green dots) or measured with an IR camera calibrated for higher temperatures (black).

More rapid and larger shifts in wavelength and temperature were expected during direct laser strikes on the sensors located at positions 4–6 on the specimen in Figure 10. Figure 13 shows results of direct strikes at position 4 (on the 1555-nm sensors) and position 6 (on the 1532-nm sensors). Figure 13a shows the wavelength shift of the front, middle and back 1555-nm sensors, plotted as blue, red and green curves, respectively, during a direct strike at position 4 at 25% laser power. A ~100 °C shift was measured in ~0.1 s in the front sensor. The temperature shift was somewhat slower in the middle and back sensors. The Bragg wavelength of the back sensor (in green) was relatively stable during the strike, similar to the response of the sensor embedded in the CF composite shown earlier in Figure 8a. In contrast, the peak Bragg wavelengths of the front and middle sensors were more erratic, since the laser energy was directly concentrated on the front surface of the specimen. At high temperatures, the peak wavelengths of these sensors were observed to hop rapidly between values during a direct strike. The Bragg wavelength of the front sensor (in blue) was indeterminate near *t* = 33 s (when no peak could be acquired by the interrogator), similar to the response in Figure 6b for a direct strike on a sensor in the GF composite, also at 25% laser power.

Though the responses in Figure 13a of the front and middle sensors (in blue and red) were more erratic during the strike than the response of the back sensor (in green), their Bragg wavelengths also clearly shifted by a larger amount. Extrapolation to higher temperatures of the vendor supplied FBG response [24] suggests that the temperatures in the composite were between ~600 °C on the back surface (for $\Delta \lambda_{B}\approx 9$ nm) and ~700 °C on the front surface (for $\Delta \lambda_{B}\approx 12$ nm). These values are well above the specified maximum temperature of 300 °C for the sensors used here. Partial erasure of the grating is expected [12], and the front and middle sensors were damaged during the strike. Their reflectivity was reduced to only 10% and 20%, respectively, whereas the reflectivity of the back sensor was approximately the same before and after the strike.

To merely detect laser radiation on the composite surface, the precise temperature is not needed. However, reliable estimates of the temperature are helpful to optimize the design of the sensor network. Since the measured sensitivity of 11.9 nm/°C was only accurate at lower temperatures [19], and the epoxy resin begins to degrade near 250 °C (burning off completely near 400 °C), an independent measurement of temperature was desired. A FLIR^®^ IR thermal imaging camera (FLIR Systems, Wilsonville, OR, USA), calibrated to higher temperatures, was placed behind the specimen to measure the thermal response on the back surface of the composite. Figure 13b shows the temperature measured by the IR camera and by the back FBG at position 6 during a direct strike on the 1532-nm sensors at 50% power. The black curve shows the temperature measured on the back surface by the IR camera. The green curve shows the estimated temperature response of the back FBG sensor, again based on an extrapolation of vendor specifications [24].

The IR camera measurement shows that the FBG sensor temperature response is roughly accurate during and after the laser strike, providing useful information about the sensor network. Both measurements in Figure 13b demonstrate that the temperature on the back surface approached 1000 °C during the strike and returned to room temperature after the strike. Similar thermal imaging tests with the IR camera during a laser strike on a six-ply CF composite [14] showed that the temperature on the back surface remained relatively steady in time (at values between 500 °C and 700 °C), depending on the incident laser power (between 30% and 70% of maximum). The shapes of the responses in the thermal imaging measurements in [14] were nearly identical to the shape of the green curve in Figure 13a.

**Figure 14:**
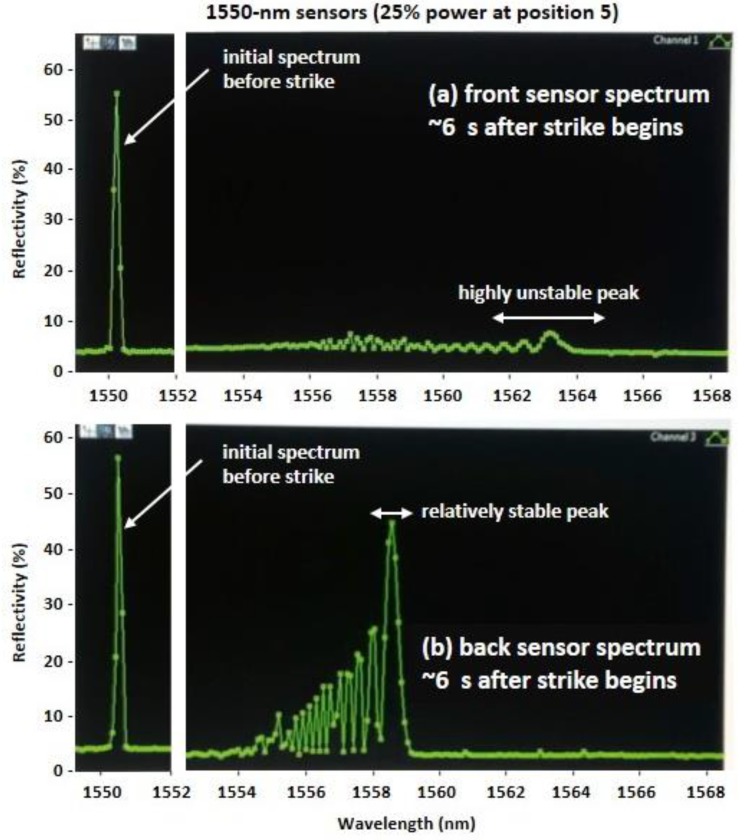
Spectral response of the (**a**) front and (**b**) back sensors (with $\lambda_{B}=1550$ nm, nominally) for a strike at position 5, initially, and ~6 s after the strike begins.

The FBG peak wavelength response in Figure 13b at higher irradiance, however, is considerably more erratic than the response measured by the IR camera. To understand the cause of fluctuations in the Bragg wavelength $\lambda_{B}$ of an FBG during a direct strike, the spectra of several sensors were observed during direct strikes at different power levels. An example of the spectrum (reflectivity vs. wavelength) is shown in Figure 14 (a and b) for the front and back 1550-nm sensors, respectively, captured ~6 s after the beginning of a direct strike at position 5 (refer to Figure 10 for location). The laser power was 25% of maximum, so the behavior of the peak wavelengths of the front and back sensors (nominally near 1550 nm) in Figure 14 should mimic the response observed in the front and back sensors (nominally at 1555 nm) in Figure 13a.

Based on the spectral responses shown in Figure 13 and Figure 14, precise acquisition of a peak wavelength in the FBG response during a direct strike on a sensor can be hindered by two effects: First, highly localized temperature spikes during a direct strike can result in a non-uniform temperature profile across the length of the sensor that can cause erratic fluctuations in the reflection spectrum and in the peak wavelength if the temperature is high enough. The resulting spectrum is similar to that caused when a non-uniform strain field is applied across the length of an FBG sensor [3,20,21,27,28]. Secondly, for direct laser strikes at higher power levels, the gratings were often erased soon after the strike began, especially for sensors embedded closer to the front surface of the composite.

Consider the spectral distortion in Figure 14, which shows one instance of a multi-peaked spectrum in the front and back sensors (nominally at 1550 nm) at a time ~6 s after the beginning of the laser strike. The distortion in this multi-peaked spectrum varied dynamically during a direct strike. For the front sensor in Figure 14a, the spectral peak with the highest reflectivity hopped rapidly between neighboring peaks [21], changing in wavelength by several nm, consistent with the behavior of the blue curve in Figure 13a. For the sensor on the back surface of the composite, shown in Figure 14b, shifts in the peak wavelength were much smaller, similar to the green curve in Figure 13a.

Given that the beam profile for the high energy laser was Gaussian with intensity $I(z)=I_{0}\exp\left(-2z^{2}/w_{0}^{2}\right)$, during a direct strike the peak irradiance $I_{0}$ occurred at position *z* = 0 (the center of the grating), and the energy was highly localized with a laser spot size $2w_{0}=5$ mm for an FBG gauge length of 10 mm. A precise explanation for the resultant spectral response in Figure 14 can be developed using a modified transfer matrix approach as described in [27], used by Prabhugoud and Peters to characterize non-uniform quadratic strain fields on strain sensors. Based on the results in [27], a transfer matrix analysis using a modified form of the grating period which is here defined consistent with Equation (4),

(5) $$\Lambda (z)=\Lambda_{0}\left[1+\left(\frac{1}{n_{e}}\frac{dn_{e}}{dT}+\alpha +(1-p_{e})\alpha_{c}\right)\Delta T(z)+(1-p_{e})\varepsilon \right],$$

would demonstrate that the spectrum in Figure 14b and the incident Gaussian intensity are consistent with the presence of a non-uniform quadratic temperature profile $\Delta T(z)≅\Delta T_{0}\left[1-{\left(z/z_{0}\right)}^{2}\right]$ across the grating length, where ε=0 in this experiment. The actual values of $\Delta T_{0}$ and $z_{0}$ would depend on the energy absorbed by the composite, its thermal conductivity, and its coefficient of thermal expansion $\alpha_{c}$.

Based on Equation (5), a non-uniform temperature profile causes non-uniform thermally-induced strain $\alpha_{c}\Delta T(z)$ for any material in which $\alpha_{c}$ is not negligible. The spectrum in Figure 14b shows that a highly localized direct laser strike results in a highly localized, dynamically varying thermal response by the sensor. In the carbon fiber/epoxy composite described by Figure 14, for which $\alpha_{c}$ is small, the dominant contribution is due to changes in the index of refraction $n_{e}$ with temperature. In the E-glass fiber/epoxy composite, described earlier in Figure 6, the non-uniform thermal response also includes thermally induced strain since $\alpha_{c}$ is markedly larger.

Finally, although the initial spectrum of the front and back sensors were very similar (a single peak near 1550 nm), the front sensor also experienced a much sharper drop in its reflectivity during the strike, as can be seen by comparing the graphs in Figure 14a,b. This is likely due to higher temperatures on the front surface than on the back surface of the carbon fiber/epoxy composite. After the strike at position 5, the peak wavelengths of both the front and back sensors returned to values close to their nominal Bragg wavelength, i.e., a single reflection peak near 1550 nm. However, the peak reflectivity of the front sensor was less than 40% after the strike, indicating partial erasure of the front sensor due to high temperature grating decay.

This suggests the second reason it is more difficult to determine a peak wavelength for an FBG sensor experiencing a direct strike. For direct strikes with a power level of 50% or greater on a two-ply composite, the gratings usually erased completely, whether on the front surface, in between the plies, or on the back surface. When this occurred, the peak wavelength measurement was indeterminate. The spectral response looked similar to that in Figure 14a, but within seconds the small peaks in the spectrum shifted to higher wavelengths (beyond the instrument measurement limit of 1568 nm), and the distorted spectrum gradually dissipated into the noise as the grating decayed completely, with no recovery of a spectral peak at the nominal Bragg wavelength. Interestingly, although the measured temperatures were sometimes quite high at laser power levels of 25%, there was often no significant damage to the sensor after the strike, permitting its reuse. Further tests would be needed to determine the accuracy of the analysis in [12] (a work describing grating decay at high temperatures) when localized thermal energy is suddenly applied to a grating under laser irradiation. These tests, however, clearly demonstrate the ability to rapidly detect temperature spikes in a composite structure using embedded FBG sensors.

## 5. Conclusions

In this study, structural health monitoring is performed using embedded FBG temperature sensors to rapidly detect laser radiation and its location on the surface of a polymer matrix composite. Rapid detection might be necessary to detect flames or to counter a directed energy attack. During a direct laser strike on a sensor, large thermal gradients of ~100 °C within ~0.1 s were detected. In sensors located 1 cm to 3 cm away from a strike, temperature shifts were also observed quickly, and they were much smaller in magnitude, producing results that indicate the proximity of the laser strike and the laser power.

Arrays of FBG sensors were also used to characterize the thermal conductivity and absorptivity of carbon fiber and E-glass fiber/epoxy composites based on their response to the localized temperature gradient caused by the laser radiation. The material-dependent response is important, for example, laser radiation was detected more rapidly by sensors embedded in a carbon fiber/epoxy composite than in an E-glass fiber/epoxy composite [19], so that fewer sensors would be needed per unit area in materials with higher thermal conductivity. The influence of convection on the temperature of the composite surface was observed only as it related to the use of exhaust and N_2_ gas (intermittently applied to suppress flames). The importance of convection as it relates to high speed airflow across a structure (such as over an aircraft wing) must be explored further.

The depth at which sensors were embedded in a composite did influence the temperature response. It was observed that FBG sensors on the front surface rapidly detected high temperatures, but could be damaged or erased as the irradiance increased. Sensors embedded beneath the front surface also detected laser radiation, with estimated temperatures approaching 600 °C during a direct strike, but with a somewhat reduced speed of detection and with considerably less damage to the sensor. Further research is needed to determine the optimal depth at which sensors should be embedded to rapidly detect higher laser power levels.

Standard FBG temperature sensors were used in this project instead of specialized high temperature FBG sensors. While high temperatures did cause damage to sensors under certain conditions, standard type I sensors were acceptable to use here since the goal was solely to detect high energy radiation as quickly as possible, before the polymer matrix composite itself was destroyed. Furthermore, even when sensors were damaged or destroyed during direct strikes at higher power levels, the initial temperature gradient could still be obtained, and neighboring sensors in the array also detected the strike.

There are conditions when high temperature FBGs may be beneficial, for example, if groups of sensors were destroyed too fast for detection to occur or if sensor functionality needed to be maintained after a strike. Results here indicate that embedding the sensor more deeply in the composite would likely alleviate these concerns. High temperature sensors would also be needed if reliable high temperature measurements were needed for instrumentation or diagnostic purposes. For example, high temperature sensors might be required to precisely characterize the quadratic temperature profile that was observed across the grating length during a direct laser strike. The non-uniform temperature profile detected at high temperatures resulted from the small spot size of the laser and the intrinsically poor thermal conductivity of the polymer matrix composite structure. At even higher temperatures, the dynamically varying spectral response caused wavelength hopping that prevented a peak wavelength interrogator from acquiring reliable values for the Bragg wavelength. A transfer-matrix analysis is likely the best approach to understand the details of such a response.

Ongoing experiments are examining the influence of strain on the detection of a localized temperature gradient [18]. Although no external mechanical strain ε was applied in the tests described here, external sources of strain that could prevent accurate identification of a localized temperature gradient ΔT must be considered. An array of sensors embedded within one or more layers of the composite may provide sufficient monitoring of the Bragg wavelength to rapidly identify both effects. Ultimately, the sensor array must be designed to ensure that the strain and thermal responses can be isolated, considering both the energy levels capable of causing damage as well as the spot size of the laser.

Signal processing algorithms that can isolate these responses due to strain and temperature are being developed. While a full spectral interrogation at a higher scan rate may be useful to provide the necessary data acquisition capability, alternative fiber optic sensing technologies are also being considered. These could include specialized FBGs that perform well at high temperatures [9] as described above, distributed optical fiber sensors based on Rayleigh scattering, or optical frequency domain reflectometry [29,30]. The insensitivity of optical fiber temperature sensors to the incoming wavelength of IR radiation is an important advantage over other techniques for sensing laser radiation on a polymer matrix composite.
